# The Effect of Dog Presence on the Therapeutic Alliance: A Systematic Review

**DOI:** 10.3390/vetsci9120669

**Published:** 2022-12-01

**Authors:** Timothy Collier, Pauleen Bennett, Vanessa Rohlf, Tiffani Howell

**Affiliations:** School of Psychology and Public Health, La Trobe University, Bendigo, VIC 3552, Australia

**Keywords:** animal-assisted therapy, psychological therapy, canine-assisted interventions, human–animal interactions, working alliance

## Abstract

**Simple Summary:**

The therapeutic alliance is the relationship between clinician and client. It is one of the most important predictors of successful treatment outcomes, regardless of the type of treatment or theoretical framework used by the clinician. Therefore, it is important to investigate the factors which may positively influence the development of a strong therapeutic alliance. One possible factor is the presence of a dog in a psychological therapy session. Dogs are known to promote positive interactions between the handler and other people, so studies have explored whether the presence of a dog during therapy positively supports the therapeutic alliance. We performed a systematic review of all research examining this topic and found six studies that met the inclusion criteria. Three of the studies found no significant effect of dog presence on the therapeutic alliance; however, the other three did find a significant effect. No studies reported any negative effects of dog presence. The reason for these conflicting results is not clear, but may relate to the types of outcome measures used, or the characteristics of the dog itself, such as breed. It is too early to recommend that dogs be employed in therapy sessions to establish a therapeutic alliance.

**Abstract:**

The development of a therapeutic alliance represents one of the most important processes that occurs in psychological therapy and is one of the strongest predictors of treatment outcome. To ensure the effective delivery of psychological interventions, it is important to explore factors which may improve the therapeutic alliance. There are well-documented effects of human–animal interactions in social settings, and researchers have also considered the effect of dog presence on the therapeutic alliance. A systematic review was conducted in accordance with the preferred reporting items for systematic reviews and meta-analyses (PRISMA) checklist. Database searches included CINAHL, Cochrane, Embase, MEDLINE, PsycINFO, and Scopus. The inclusion criteria were studies that assessed the effect of dog presence on the therapeutic alliance and provided a quantitative outcome measure. Six studies met the inclusion criteria and were included in the systematic review. Three of the included studies observed no significant effect of dog presence on the therapeutic alliance; three studies did observe a positive effect, with effect sizes ranging from d = 0.10 to d = 0.58. All six studies took place in either research or clinical settings. Studies differed in terms of help-seeking versus non-help-seeking populations, where help-seeking populations were genuinely pursuing a psychological intervention. Heterogeneity was observed regarding study procedure and outcome measures used. Current data is limited, and initial evidence suggests that the effect of dog presence on the therapeutic alliance remains unclear, illustrated by inconsistent outcomes across the included studies. Further research is warranted before introducing dogs into therapeutic settings for this purpose.

## 1. Introduction

The therapeutic alliance is considered to be one of the most important components of psychological therapy. Regardless of the intervention or theoretical framework, research has consistently placed the therapeutic alliance as one of the best overall predictors of treatment outcome [1]. In contrast, a delay in developing, or failure to develop, the therapeutic alliance is associated with poor treatment outcomes, including lack of symptom change, a reduction in future help-seeking, and a negative view of the psychological profession [2]. Classically defined by Bordin [3], the therapeutic alliance consists of three parts: a relational bond between clinician and client; agreement on the goals of treatment; and agreement on the tasks of psychological therapy. In order for the alliance to develop positively, Bordin [3] contended that the therapeutic engagement must be underscored by each component.

A number of studies have considered clinician-based factors that contribute to the development of a clinician–client bond. In a literature review, Horvath [4] suggested that interpersonal and intrapersonal processes are fundamental to the development of this bond. Interpersonal processes involve: clear and open communication; conveying understanding; having the ability to assess the relationship from the client’s perspective; and, communicating with language that is supportive and respectful [4]. Additionally, it includes the clinician’s capacity to: express sensitivity; maintain awareness of the client’s need for a psychologically safe environment; maintain a sense of hope; and, respond to challenges appropriately [4].

Alongside interpersonal processes, Horvath [4] contended that intrapersonal, clinician-based processes are also important. This includes the “specific psychological make-up of the therapist” [4] and the subsequent dynamics that arise as a result of this [4]. While researchers have considered how to support early career psychologists in developing interpersonal skills, outcomes suggest that trainability in this area is possible (e.g., deliberate practice), but may be limited [5]. As a result, research has shifted to considering whether there are features beyond clinician-based factors that may impact the development of the therapeutic alliance.

There are well-documented effects of human–animal interactions in social settings. Beetz, Schöfmann [6] suggested that humans across the lifespan “communicate more, and more positively, verbally and non-verbally, in the presence of friendly animals” [6]. Additionally, people that are accompanied by a friendly looking animal receive more positive attention, and are perceived as being more trustworthy by others [6]. These outcomes may occur due to animals facilitating social interaction by acting as ice-breakers during initial engagements [7]. Studies have investigated this effect in domestic dogs, and observed that dog presence facilitates an increased number of spontaneous conversations with strangers across a variety of settings [8,9]. 

While research generally indicates a positive effect of dog presence on social interactions, Wells [10] argued that the social catalysis effect depends on the individual dog. In an observational study, the author recorded the behaviour of 1800 pedestrians approaching an experimenter on a city street who was accompanied by either a Labrador Retriever pup, a Labrador adult, a Rottweiler adult, or one of two neutral stimuli (i.e., teddy bear or potted plant). She observed that more people ignored the experimenter when they were alone or with the neutral stimuli. However, the presence of the Rottweiler resulted in more non-responses (i.e., no response, no smile, or no conversation) compared to the puppy or adult Labrador [10]. Consequently, it was concluded that certain dog breeds are poorer at facilitating social interactions [10]. This outcome was consistent with earlier research by McNicholas and Collis [9] who subsequently hypothesised that breeds with a reputation for being aggressive may not serve as social catalysts when compared to those with a more favourable reputation.

Research in therapeutic settings has established that the presence of a dog can reduce anxiety, encourage interaction between people, and enhance the way in which people are perceived [11]. These effects of dog presence may translate directly to aspects of the therapeutic alliance, and, theoretically, should enhance them. However, outcomes from individual research studies investigating the effect of dog presence on the therapeutic alliance are inconsistent. A systematic analysis of available literature is therefore warranted to develop our current understanding in this area of research. A systematic review and, should the data permit, a meta-analysis, may add depth to existing literature by providing a synthesis of relevant research and increasing our understanding of whether a reliable effect of dog presence is observed across studies. This understanding may inform clinical practice by providing clinicians with stronger evidence to support decision making regarding the presence of dogs in psychological settings. The aim in this study was to collate available literature, using a systematic review and, if appropriate, a meta-analysis, to evaluate whether dog presence has an effect on the therapeutic alliance.

## 2. Materials and Methods

A systematic review was conducted in accordance with the preferred reporting items for systematic reviews and meta-analyses (PRISMA) checklist [12]. 

### 2.1. Search Method

A literature search was conducted using the following databases: CINAHL, Cochrane, Embase, MEDLINE, PsycINFO, and Scopus. Relevant search terms associated with the research question were combined using Boolean operators as follows: “therap* alliance” OR “therap* relationship*” OR “therap* bond*” OR “working alliance” OR “client* perception*” OR “alliance” AND “dog*” OR “canine*” OR “animal-assisted”. The search was conducted on Saturday 23 July 2022.

### 2.2. Inclusion and Exclusion Criteria

The following inclusion criteria were used for the systematic review:

1. Include if
  - published in a peer-reviewed journal written in English, with no restrictions on year of publication;
  - dissertation or thesis written in English, with no restrictions on year of publication
  - participants were adults;
  - the study examined dog-presence;
  - the study examined the therapeutic alliance using a quantitative measure, with or without standard therapy;
  - participants engaged directly or indirectly in a simulated therapeutic experience.
2. Exclude if
  - the study was a systematic review, meta-analysis, or a narrative review;
  - the study was an editorial, conference abstract, letter, or case report;
  - the study was a dissertation or thesis where any subsequent peer-reviewed journal publication arose from the original work.

### 2.3. Study Selection

Articles were assessed for inclusion using a two-stage approach: title and abstract screening, and full text screening. Covidence systematic review software (Covidence, Melbourne, VIC, Australia; covidence.org) was used to manage screening and data extraction. Two authors (TC and VR) independently appraised the studies for inclusion or exclusion. Each reviewer was blind to the decision of the other reviewer when conducting both stages of the screening process.

Articles with relevant titles were selected for inclusion if they contained the words ‘therapeutic alliance’ OR ‘working alliance’ AND ‘dog’ OR ‘canine’. Articles with relevant abstracts were selected for inclusion if they indicated that the article was: an empirical study; written in English; used human participants; and, referred to the therapeutic alliance and the presence or use of a dog.

Articles were included if full-text screening indicated that participants engaged in a direct or indirect therapeutic experience (e.g., simulated clinical interview) where a dog was present, and the therapeutic alliance was quantitatively measured. Articles that did not meet these criteria were excluded.

There was some confusion regarding a study by Wesley et al. [13]. The statistical analysis yielded identical results to an earlier study published by Minatrea and Wesley [14]. An attempt to contact the authors was made to clarify whether the populations used in these studies were discrete, but it was unsuccessful. Consequently, the decision was made to exclude the study by Minatrea and Wesley [14] and include the study by Wesley et al. [13], because they included more detail regarding the methodology.

### 2.4. Data Extraction

The following data were extracted by TC: study design; population; methodology; type of dog; measures of the therapeutic alliance; and quantitative outcomes.

#### Quality and Risk of Bias Assessment

The JBI critical appraisal checklist for quasi-experimental studies [15] was used to assess the quality and risk of bias of included studies. This tool was chosen as it has the capacity to assess a broad range of quasi-experimental designs [15]. Quality assessment was performed by TC. A second reviewer (VR) was consulted when further discussion regarding a particular study was warranted. Studies were assessed for selection and comparison of study groups, outcome measurement, statistical analysis, and overall methodological rigour.

### 2.5. Data Analysis

A narrative synthesis of the findings from the included studies was collated in Excel. Reference was made to study design, population type, methodology of study, dog characteristics, quantitative outcome measures, and qualitative outcomes. Data were assessed for homogeneity to determine the feasibility of a meta-analysis. Due to the observed heterogeneity of study designs, included populations, and outcome measures, undertaking a meta-analysis of the data was not feasible. Therefore, only the results of the systematic review are presented in the remainder of this manuscript.

## 3. Results

**Figure 1:**
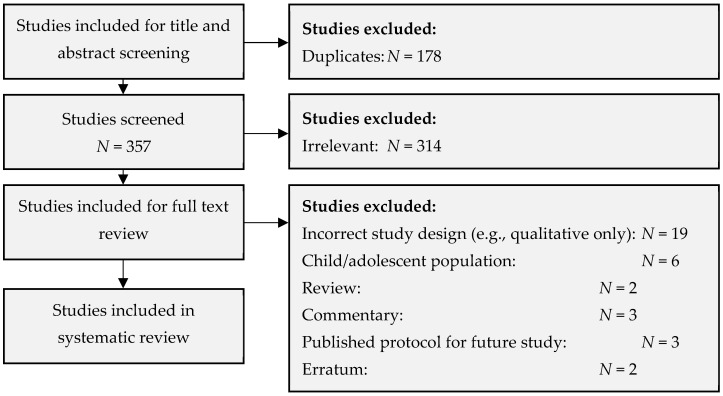
PRISMA flow diagram for the database search and review process.

The initial search returned a total of 535 studies. Following removal of duplicates and subsequent assessment against inclusion and exclusion criteria, a total of six studies were included in this review. This is illustrated in the PRISMA flow diagram in Figure 1.

### 3.1. Included Studies

#### 3.1.1. Study Design

All six included studies used quasi-experimental post-test only designs, as shown in Table 1 [6,11,13,16,17,18].

#### 3.1.2. Population

Three of the included studies sampled non-help-seeking students from United States and/or Canadian universities [11,16,17]. One study sampled help-seeking students from a United States university [18]. One study sampled participants who met criteria for substance dependency [13]. The most recent study sampled United States military soldiers with a diagnosis of post-traumatic stress disorder [6].

#### 3.1.3. Procedures

Procedures ranged from simple dog presence through to the inclusion of a dog as part of a formal animal-assisted psychological intervention (AAI), in which the dog actively participated in the intervention via specific behaviours [13]. Similarly, the included studies varied in duration, with most studies assessing a single interaction between the participant and the interviewer. The exception to this was one study that assessed US military soldiers three-hours per week over four-weeks as part of an AAI-type program [6]. The study by Beetz, Schöfmann [6] included a control condition that engaged in a non-equivalent comparison compared to the experimental condition. The experimental condition engaged in both individual and group-based treatment, where a dog was present for the group component. In contrast, the control condition engaged in individual treatment only.

Three of the included studies assessed the therapeutic alliance by engaging participants in an interview that simulated an initial psychological therapy session [16,17,18]. One study involved participants engaging in a one-hour group therapy protocol [13], and one asked participants to view a video recording of a simulated therapeutic interaction [11].

#### 3.1.4. Dog Characteristics

The dogs in the studies varied by breed, age, and training experience. Across the six included studies, the following breeds were used: Labrador Retriever; Belgian Malinois; Cavalier King Charles Spaniel; Beagle Cross; and Collie/Labrador Cross. The age of the dog ranged from one to eight years old. Similarly, each dog had a different level of training and experience. For example, some dogs had previous experience working in therapeutic settings, while others had less or nil therapeutic experience, but a considerable amount of behavioural training. In most cases, the dogs were owned by members of the research project. The exception to this was the study by Beetz, Schöfmann [6], who used assistance dogs from a military-based dog handling school. It was unclear whether the assistance dogs had experience in therapeutic settings.

#### 3.1.5. Measures of the Therapeutic Alliance

Two of the included studies used the Counsellor Rating Form–Short Version (CRF-S) to measure the therapeutic alliance [11,16]. Two used the Working Alliance Inventory (WAI) [17], and one used the Helping Alliance Questionnaire (HAQ-II) [13,18]. One of the included studies self-developed a set of questions that related to evaluating the therapeutic relationship using a five-point Likert scale [6].

#### 3.1.6. Quantitative Outcomes

Three of the included studies observed no significant effect [6,16,18]. Three studies observed a significant effect [11,13,17]. Two of these studies reported effect sizes, illustrated in Table 1 [13,17]. Effect sizes ranged from small (r = 0.10) [13] to moderate (r = 0.58) [17].

### 3.2. Quality and Risk of Bias

The quality and risk of bias assessment is summarised in Table 2. All six of the identified studies were considered to be of suitable quality for inclusion in the systematic review. Similarly, risk of bias for all identified studies was indicated to be low. Generally, the appraisal was highly consistent across the included studies. However, the study by Beetz et al. [6] utilised a self-developed measure of the therapeutic alliance. Data regarding internal reliability were included in the publication, but it is not yet a validated measure of the therapeutic alliance. 

## 4. Discussion

This review systematically evaluated whether dog presence has an effect on the therapeutic alliance. The effect remains unclear due to inconsistent outcomes across the included studies. Specifically, three of the reviewed studies observed positive effects, evidenced by significantly increased scores on the therapeutic alliance measure for the experimental condition compared to the control condition. In contrast, three of the reviewed studies did not observe a significant effect. Consequently, current evidence does not support the presence of a robust relationship between these variables, although the potential for harm seems minimal, due to no studies reporting any negative effects of the intervention. An inconclusive outcome is relatively unsurprising, given the lack of homogeneity observed across studies with respect to population, procedure, dog characteristics, and outcome measures. However, all included studies were considered to be acceptable regarding quality and risk of bias, so these factors are unlikely to explain the inconsistency in outcomes.

### 4.1. Factors Potentially Contributing to Heterogeneity in Outcomes

#### 4.1.1. Outcome Measures

The measures used to evaluate the therapeutic alliance in the included studies were inconsistent. This represents a challenge to interpreting research outcomes in this area. While the WAI and HAQ-II are well-established measures of the therapeutic alliance, the CRF-S may not be the most appropriate measure for this research. While this measure addresses themes of the therapeutic alliance, it was designed to assess attributes of the clinician, rather than the therapeutic relationship more broadly [19]. Moreover, the subscales of worthiness, expertness, and attractiveness are generally inconsistent with the theoretical framework proposed by Bordin [3], which emphasises the bond between clinician and client, agreement on the goals of treatment, and agreement on the tasks of psychological therapy. Consequently, the outcomes described by Goldman et al. [16] and Schnieder and Harley [11] were less relevant when compared to studies that utilised the WAI or HAQ-II. Similarly, the self-developed questionnaire used by Beetz, Schöfmann [6] is not yet a validated measure of the therapeutic alliance. Consequently, the outcomes reported have merit, but are less impactful than the other included studies.

Approaches to measuring the therapeutic alliance appear to be inconsistent when comparing research studies to clinical practice. In clinical practice, the use of measures such as the HAQ-II and WAI are uncommon [20]. This may be explained by their complexity and length of administration [20]. Instead, the Session Rating Scale [20] is arguably the most widely adopted measure of the therapeutic alliance in clinical practice, due to its brief, four-item visual analogue format [21]. The SRS is adapted from initial work by Bordin [3], with an additional emphasis on the client’s theory of change, as described by Gaston [22]. In this theory, Gaston [22] underscores the importance of congruence between the clinician and client’s beliefs about how people change in therapy. Duncan et al. [20] provided evidence that the SRS has strong internal and external reliability, as well as concurrent validity with the HAQ-II, so the SRS represents an appropriate measure for use in both research and clinical practice. As such, it is recommended that future studies consider evaluating the therapeutic alliance with this measure. Pragmatically, the measure is briefer, which will reduce burden on the participant, without compromising reliability or validity. The use of this measure will create consistency between clinical and research settings, supporting the translation of any future research outcomes to clinical practice, while minimising measurement variability across studies.

A broader challenge to evaluating the therapeutic alliance is the ipsative design of current measures. Such designs make it difficult to understand whether a particular intervention is having a negative effect on the alliance. While none of the included studies reported any negative effects of the intervention, this cannot be empirically evaluated by current measures.

#### 4.1.2. Study Populations and Procedures

Across the included studies, a variety of participant populations and study procedures were employed. Three studies included participants from clinical, help-seeking populations, while the other three studies investigated effects in US or Canadian undergraduate students who were non-help-seeking. While qualitatively this did not appear to have an effect on outcomes, the theoretical basis of the therapeutic alliance is underscored by the collaborative development of goals and tasks of psychological therapy [3]. Consequently, if participants are not genuinely help-seeking, evaluating these aspects of the alliance is not feasible.

With respect to procedure, the approach by Cieslak [18] appeared to be the most robust. In comparison to other included studies that adopted a single-session interview paradigm, Cieslak [18] recruited participants who were genuinely help-seeking. Additionally, the 50-minute clinical interview was completed using a combination of structured and unstructured questions with unrehearsed responses. Compared to the methodology adopted by Goldman et al. [16], who used a brief, highly structured interview format with scripted therapeutic responses, the approach by Cieslak [18] may be more representative of clinical practice. As discussed, the study by Beetz, Schöfmann [6] included a control condition that engaged in a non-equivalent comparison compared to the experimental condition. Consequently, measurement of the therapeutic alliance may have been confounded by the effect of the group-based intervention. 

For all included studies, it was unclear whether a therapeutic approach that emphasised the collaborative development of goals, tasks, and approach to treatment was used. If these aspects of the approach were not adequately incorporated into the intervention, the development of the therapeutic alliance may have been adversely impacted, or perhaps, fundamentally unable to develop. If this occurred, dog presence would be unlikely to enhance a limited or non-existent alliance.

#### 4.1.3. Dog Characteristics

Dog characteristics are a potential confound that were not addressed by the included studies. Variable dog characteristics included breed, size, colour, and subjective perception (e.g., friendliness) [23]. Each of these characteristics may partly contribute to the mixed outcomes observed in this review. For instance, a study by Woodward et al. [23] observed that breed-specific differences were the most powerful predictors of interpersonal trait attributions (e.g., dominant, submissive, hostile, friendly). Specifically, they found that, regardless of colour, certain breeds have less positive personality stereotypes associated with their image [23]. These breeds included the standard Poodle, German Shepherd, Boxer, and Rottweiler [23]. The size of these effects was large, ranging from *r* = 0.92 to *r* = 0.97 across the trait categories. Notably, the present systematic review included one study where a Belgian Malinois was present for parts of the intervention [6]. This breed shares similar characteristics with the German Shepherd, and may be subject to equivalent stereotypes described by Woodward et al. [23]. While these breeds have interpersonal qualities that may make them suitable for work in therapeutic settings, people can sometimes hold negative biases towards these dogs. Consequently, these biases may be subtly reflected in the relationship with the clinician, and negatively affect the therapeutic alliance.

### 4.2. Theoretical Implications

It was theorised that, due to dogs having a social catalysis effect on social interactions, dog presence should improve the relational bond between clinician and client. Specifically, dog presence should enhance interpersonal processes by acting as an ice-breaker during interactions between the clinician and client. As discussed, the relational bond represents a fundamental part of the therapeutic alliance, as defined by Bordin [3], and the reviewed literature underscores the importance of interpersonal and intrapersonal processes in the development of this bond [4]. Due to the mixed outcomes observed across the included studies, it is too early to conclude whether or not there is support for this theory. However, the literature indicates that the appearance of the dog is an important factor with respect to the social catalysis effect [10], whereby certain breeds have an increased capacity to elicit this effect [10]. In this systematic review, a variety of breeds were included. While most of them may generally be perceived as friendly (e.g., Labrador Retriever, Golden Retriever, Beagle), the Belgian Malinois present in the study by Beetz, Schöfmann [6] may have been perceived as less friendly, at least by some participants. This study did not observe a positive effect of dog presence, and breed may have contributed to this. However, due to the limited number of included studies, it is difficult to realistically evaluate the secondary effect of breed on the observed outcomes. It is pertinent for future research to consider the potential effect of breed, as, theoretically, the social catalysis effect intrinsically relies upon the dog being perceived as friendly. Consequently, if the dog is not perceived to be friendly, it is unlikely to positively influence social interactions, thus having limited effect on the relational bond between clinician and client.

### 4.3. Strengths and Limitations

To our knowledge, this systematic review represents the first attempt to critically evaluate and integrate research investigating the impact of dog presence on the therapeutic alliance in psychological settings. A strength of this review is that it underscores the heterogeneity in research design among existing research studies. Variability was observed with respect to population, procedure, dog characteristics, and outcome measures, and this review provided pragmatic recommendations to address these concerns in future studies.

This review included a relatively low number of studies, reflecting a paucity of research in this area. Perhaps more importantly, no randomised controlled trials were included in the analysis. This outcome is reflective of broader research investigating animal-assisted interventions in psychological settings (e.g., Meixner and Kotrschal [24]), and raises concern regarding the scientific standards in human–animal research. While this is a weakness of this area of research, implementing a randomised controlled trial design has salient challenges. Most obviously, it is impossible to blind participants to dog presence. Additionally, it is not feasible to measure the therapeutic alliance before an interaction with the clinician or interviewer. Consequently, a pre-intervention/baseline evaluation of the alliance is not possible using current research paradigms. However, this is an emerging area of research, and these limitations represent opportunities for future studies to build on.

A possible alternative for researchers to consider is a paradigm that allows for a more robust initial evaluation of the therapeutic alliance as part of the intervention. For example, a future study may recruit participants that are genuinely help-seeking. This may be achieved in universities via campus counselling and/or psychology clinics. Then, participants are randomised to either the experimental (i.e., dog present) or control (i.e., no dog present) condition. Participants complete a 90-minute initial psychological assessment (i.e., clinical interview with structured and unstructured components, ensuring goals, tasks, and approach are discussed) in two phases. In the first phase, the participant and clinician/interviewer engage in the assessment for 45-minutes. The assessment is then paused for five-minutes, and the participant completes the Session Rating Scale. In the second phase, both conditions complete an additional 45-minutes of the assessment. However, in this phase, a dog is present in the experimental condition, for half the participants. The dog is briefly introduced to the participant, and the participant is invited to pet the dog. The dog then sits in a neutral position between the participant and clinician/interviewer while the assessment continues. At the conclusion of this phase, all participants complete the Session Rating Scale for a second time, including those who had a dog in the room during the second half of the assessment, and those who did not.

This approach represents a shift from simulated interventions to research that is fundamentally naturalistic in the context of design and duration. This is necessary, given that the central principles and theory of the therapeutic alliance are constructed on genuine therapeutic relationships. Furthermore, this approach ensures that potential between-group differences at baseline are considered. The analyses can then compare pre- versus post-intervention scores of the therapeutic alliance between groups. If this methodology is adopted alongside appropriate participant randomisation, the completion of a robust, randomised controlled trial is feasible. Transitioning to this level of evidence would add significant value to this area of research by establishing more robust outcomes. It is recognised that there are salient challenges to conducting this type of research. For example, pausing during the clinical interview to engage in the research component of the session may be ethically challenging, particularly when working with populations who are help-seeking. Additionally, individuals who do not like dogs, are afraid of dogs, or are allergic to dogs should not be asked to participate.

### 4.4. Clinical Implications and Future Research

A number of salient clinical implications can be generated as a result of this systematic review. Firstly, in psychological settings, there is currently unsatisfactory evidence to support the role of dog presence in establishing a therapeutic alliance. However, an increasing number of clinicians are incorporating dogs into their psychological work in Australia [25]. It is important that clinicians recognise that, for the purposes of building an alliance, current evidence remains unclear regarding the effect of dog presence in this process. Theoretically, it is appealing to endorse dog presence in psychological settings, as research suggests that dogs enhance communication and feelings of trustworthiness towards the handler [6]. Rationally, these effects should be observed between clinician and client too, and be reflected in the therapeutic alliance. However, while some of the studies included in this review observed positive effects on the alliance, it is still too early to say whether the presence of a dog is beneficial to the therapeutic alliance. Consequently, further research is warranted before introducing dogs into therapeutic settings for the purpose of supporting the therapeutic alliance. It would be useful to research whether the mere presence of a dog is sufficient, or whether specific types of interactions are necessary. 

Secondly, few of the studies included in this review provided strong rationale regarding their selection of dog [but see 13], with one study not reporting any characteristics (including breed) [17]. Future studies should consider the potential effect of dog characteristics when selecting a dog for use in this research area and provide rationale for their selection. Importantly, this may vary in different contexts, so it must be considered carefully. Future studies should report the breed of dog, as well as relevant information regarding behavioural characteristics, training, and experience.

Finally, if clinicians continue to use dogs in clinical practice, the animal’s welfare must be adequately considered. While animal welfare in this context is an emerging area of research, a review by Glenk [26] indicates that consideration of the physical environment; the frequency and duration of appointments; and the age and familiarity of individuals engaging with the dog contribute to animal welfare indicators (e.g., cortisol levels, heart rate, and observable behaviour). Additionally, d’Angelo, d’Ingeo [27] suggest that transportation can be a significant stressor, and deserves particular care. Collectively, appropriate consideration of these factors is essential to ensuring that the welfare of dogs in psychological settings is maintained.

## 5. Conclusions

In sum, it is too early to conclude whether dog presence has an effect on the therapeutic alliance in psychological settings, although none of the included studies reported any adverse outcomes. Current evidence does not support the presence of a robust relationship between these variables, with studies reporting mixed outcomes, including three studies reporting positive effects and three studies finding no effects. Finding a negative effect is currently not possible with existing validated measures of the therapeutic alliance. Considering the broader effects of dog presence on interpersonal processes in non-clinical settings, it is surprising that more studies have not observed positive effects in therapeutic-based interactions. However, given the observed heterogeneity in population, procedure, dog characteristics, and measurement of the therapeutic alliance, inconsistent outcomes are to be expected. Indeed, one might reasonably question whether several of the available studies provided a context in which a therapeutic alliance might be expected to form, in which case measuring the effect of a dog on this putative alliance is nonsensical. The development of a therapeutic alliance represents one of the most important processes that occurs in psychological therapy and is one of the strongest predictors of treatment outcome. Consequently, the continued exploration of factors that may improve the alliance remain important to the delivery of effective psychological interventions. We hope that the recommendations in this review can encourage researchers to continue to evaluate the relationship between dog presence and the therapeutic alliance by transitioning to novel research paradigms based on naturalistic designs.

## Figures and Tables

**Table 1:** Summary of Data Extraction for Included Studies.

Study	Population	Methodology	Type of Dog	Outcome Measure	Quantitative Outcomes
Beetz et al. [6]	N = 29 help-seeking adult US military soldiers with diagnosis of PTSD (three female; 26 male); average age = 38 years	Assigned to one of two conditions: group-based psychological therapy with dog; individual psychological treatment without dog. In the dog condition, participants engaged in four, three-hour group sessions over a four-week period. In the dog absent condition, participants engaged in individual psychological treatment	Labrador Retriever or Belgian Malinois	Self-developed set of nine questions regarding the therapeutic relationship; five-point Likert scale where higher score indicates a stronger relationship	Non-significant effect
Cieslak [18]	N = 30 help-seeking US undergraduate students (18 female; 12 male); average age = 20.5 years. Excluded participants with possibility of personality disorder	Assigned to one of two conditions: interview with dog; interview without dog. In each condition, participants engaged in a 50-minute clinical interview	Labrador Retriever	Working Alliance Inventory (WAI)	Non-significant effect
Goldmann, Hatfield [16]	N = 71 non-help-seeking US undergraduate students (55 female; 16 male); average age = 18 years	Assigned to one of two conditions: interview with dog; interview without dog. In each condition, participants engaged in a 10- to 15 min scripted interview that simulated a clinical interview	Cavalier King Charles Spaniel	Counsellor Rating Form-Short Version (CRF-S)	Non-significant effect
Schneider and Harley [11]	N = 85 non-help-seeking Canadian undergraduate and graduate students (51 females; 34 males); average age = 26 years	Assigned to one of four conditions: male therapist with dog; female therapist with dog; male therapist without dog; female therapist without dog. In each condition, participants viewed a video recording of a therapist introducing themselves	Golden Retriever or Collie/Labrador Cross	Counselor Rating Form—Short Version (CRF-S)	Significant effect. No effect size reported
Thomas [17]	N = 82 non-help-seeking US undergraduate psychology students (49 female; 33 male); average age = 18.48 years	Assigned to one of two conditions: interview with dog; interview without dog. In each condition, participants engaged in a structured clinical interview of unknown duration	Not reported	Working Alliance Inventory (WAI)	Significant effect; moderate effect size, *d* = 0.58
Wesley, Minatrea [13]	N = 231 help-seeking adults with diagnosis of substance dependency (117 females; 114 males); 50% of population under 25 years	Assigned to one of two conditions: group-based psychological therapy with dog; group-based psychological therapy without dog. In each condition, participants engaged in one, 60-min session	Beagle Cross	Revised Helping Alliance Questionnaire (HAQ-II)	Significant effect; small effect size, *d* = 0.10

**Table 2:** Summary of quality and risk of bias for the included studies.

Appraisal Checklist	Study					
	Beetz et al. [6]	Schneider and Harley [11]	Wesley, Minatrea and Watson [13]	Goldmann, Hatfield and Terepka [16]	Thomas [17]	Cieslak [18]
Is it clear in the study what is the ‘cause’ and what is the ‘effect’ (i.e., there is no confusion about which variable comes first)?	Yes	Yes	Yes	Yes	Yes	Yes
Were the participants included in any comparisons similar?	Yes	Yes	Yes	Yes	Yes	Yes
Were the participants included in any comparisons receiving similar treatment/care, other than the exposure or intervention of interest?	No	Not applicable	Yes	Yes	Yes	Yes
Was there a control group?	Yes	Yes	Yes	Yes	Yes	Yes
Were there multiple measurements of the outcome both pre and post the intervention/exposure?	No. Post-test only	No. Post-test only	No. Post-test only	No. Post-test only	No. Post-test only	No. Post-test only
Was follow up complete and if not, were differences between groups in terms of their follow up adequately described and analysed?	Not applicable	Not applicable	Not applicable	Not applicable	Not applicable	Not applicable
Were the outcomes of participants included in any comparisons measured in the same way?	Yes	Yes	Yes	Yes	Yes	Yes
Were outcomes measured in a reliable way?	Yes, but validity is unclear.	Yes	Yes	Yes	Yes	Yes
Was appropriate statistical analysis used?	Yes	Yes	Yes	Yes	Yes	Yes
**Overall appraisal**	**Include**	**Include**	**Include**	**Include**	**Include**	**Include**

## Data Availability

Not applicable.
